# Proteomic analysis of adipose tissue revealing differentially abundant proteins in highly efficient mid-lactating dairy cows

**DOI:** 10.1038/s41598-022-13964-x

**Published:** 2022-06-13

**Authors:** Yehoshav A. Ben Meir, Jayasimha R. Daddam, Gitit Kra, Hadar Kamer, Yuri Portnick, Yishai Levin, Maya Zachut

**Affiliations:** 1 Department of Ruminant Science, Volcani Center, Institute of Animal Sciences, Agricultural Research Organization, Rishon LeTsiyon, Israel; 2 The Nancy and Stephen Grand Israel National Center for Personalized Medicine, Weizmann Institute of Science, 7610001 Rehovot, Israel

**Keywords:** Immunology, Metabolism, Fat metabolism, Proteomics, Proteomic analysis

## Abstract

The improvement of nutrient utilization efficiency in dairy cows represents an important task in view of the current rising demand for animal products and sustainable resource usage. In this perspective, the identification of appropriate markers to identify the most efficient animals for dairy production becomes a crucial factor. Residual feed intake (RFI), which represents the difference between predicted and actual intake, is used to define the efficiency of cows. In this study, subcutaneous adipose tissue (AT) was collected from five high efficient (HEF) and five low efficient (LEF) mid-lactation Holstein dairy cows, that represented subgroups of the 20% lowest RFI values (HEF) and highest 20% RFI values (LEF), out of a cohort of 155 cows that were examined for feed efficiency at the individual dairy barn at Volcani Institute, Israel. Adipose samples were examined for proteomic analysis by nano-LC/MS–MS and gene expression by RT-PCR. A total of 101 differential proteins (*P* ≤ 0.05 and fold change ± 1.5) and two protein networks related to feed efficiency were found between HEF and LEF cows. Among the enriched top canonical pathways, FAT10 signaling, EIF2 signaling, Sirtuin signaling, Acute phase response signaling, Protein ubiquitination and mTOR signaling pathways were related to feed efficiency in AT. Furthermore, abundance of transferrin (TF; FC = 78.35, *P* = 0.02) enriched pathways, including mTOR signaling, LXR/RXR and FXR/RXR activation was found in AT of HEF cows. Relative mRNA expression of *RBM39*, which is involved in energy metabolism, was decreased in AT of HEF versus LEF. The relationship found between the AT proteins and/or metabolic pathways and the feed efficiency demonstrates that AT may reflect metabolic adaptations to high efficiency, and suggests that these proteins together with their metabolic mechanisms are suitable candidates as biomarkers to identify efficient cows for dairy production.

## Introduction

Residual feed intake (RFI) is measured by energy expenditure and efficiency in converting energy into body weight gain, which is related to animal feed efficiency (FE)^1^. RFI is calculated by the difference between actual feed intake and the expected feed according to their milk yield, body weight, and days in lactation (DIM). Inefficient animals with high RFI eat more than predicted based on their weights and production rate, whereas efficient animals with low RFI eat less. For a given production rate and animal size, this measurement examines the large individual diversity in feed intake. In cattle^2,3^, there is large variation in RFI values, and related studies have studied at the molecular mechanisms underlying FE-related traits. With rising demand for animal protein and the need for sustainable resource usage, increasing nutrient utilization efficiency is critical for the viability of animal production. Understanding the biology of FE in dairy cows would enable the improvement of biomarkers for identifying and selecting the most efficient animals for livestock production. The utilization of feed by animals is a complex biological process that varies depending on a variety of factors like the type and amount of feed intake, breed, sex, and environmental factors^4,5^.

The adipose tissue (AT) is a complex organ that, in addition to being the key organ in energy metabolism, serves as a critical component of many essential metabolic processes. Studies of AT proteomes provide for a better understanding of the organ's function as well as the biochemical and physiological elements of animal metabolism in general^6^. In this way, using a proteomic approach to study AT can help us learn more about the molecular interactions that determine FE and help to identify markers in efficient cattle and improve productivity.

During the last few decades, the AT has been known as an important endocrine tissue capable of producing and secreting adipokines or adipocytokines^7,8^. These include polypeptides as well as non-protein molecules, which are active metabolic factors playing a role in a variety of physiological activities such as glucose and lipid metabolism, angiogenesis, immunological function, and reproductive functions in humans and animals^9^. In cattle, the identification of genes, peptides, and networks potentially linked to FE has been a focus, with lipid, protein, and energy metabolism, immunological response, signaling pathways, and ions transport being often related to FE^10^. Transcriptomic analysis has been widely used in different tissues to relate gene expression and gene interaction networks to FE^11–22^. Previous research on cows’ AT around the time of parturition revealed an abundance of different biomarker proteins linked to insulin resistance^23^, and biomarkers related to heat stress in AT of late-pregnant cows were identified using the proteomic approach^24^. However, little is known regarding the relationship between AT proteins and metabolic mechanisms, and specifically FE, in mid-lactation dairy cows. The purpose of the present study was to compare the proteomic profile of proteins identified in the subcutaneous AT of high FE (HEF, within the lowest 20% RFI of the examined cows) and low FE (LEF, within the highest 20% RFI), and to analyze the protein networks related to FE pathways in the AT of dairy cows.

## Results

### Performance and blood parameters

In the present study, ten mid-lactating dairy cows (on average 110 ± 14 DIM, average lactation number 2.4 ± 0.5) were retrospectively divided into HEF and LEF according to their RFI values. By definition, RFI (kg/d) was significantly lower (− 0.59 and 4.80, SEM, *P* < 0.01; Table 1) for HEF than in LEF cows. In addition, FE was measured by the ratio of energy corrected milk (ECM) divided by dry matter intake (ECM/DMI), and it was higher in HEF than LEF group (*P* < 0.01; Table 1). Averages of milk, ECM, milk component yield, body weight, average daily gain (ADG) and body condition score values of the HEF and LEF groups are presented in Table 1.

**Table 1:** DM intake, milk production and efficiency of high and low efficient cows.

	High efficient (HEF)	Low efficient (LEF)	*SEM*	*P*-value
n	5	5		
DMI, kg/d	26.2	31.1	1.72	0.18
ECM, kg/d	40.4	37.1	2.49	0.25
RFI, kg/d	− 0.59	4.80	1.02	0.01
ECM/DMI	1.55	1.19	0.07	0.01
Milk, kg/d	50.3	44.5	2.92	0.33
Milk fat, %	2.99	3.11	0.10	0.79
Milk protein, %	2.86	3.09	0.06	0.05
Milk lactose, %	4.86	4.96	0.04	0.27
BW, kg	575.4	610.7	27.2	0.37
ADG, kg/d	0.29	0.35	0.08	0.98
Body condition score, units	2.6	2.5	0.06	0.49

In plasma samples, no difference in the average concentrations of insulin (1477.8 and 1521.3 pg/ml in HEF and LEF, respectively, SEM = 177.9, *P* = 0.77), glucose (71.2 and 67.4 mg/dL in HEF and LEF, respectively, SEM = 2.07, *P* = 0.26) and beta hydroxybutyrate (BHBA; 7.2 and 7.2 mg/dL in HEF and LEF, respectively, SEM = 0.3, *P* = 0.26) were detected between groups.

### Quantitative proteomic analyses of AT from HEF and LEF

**Figure 1:**
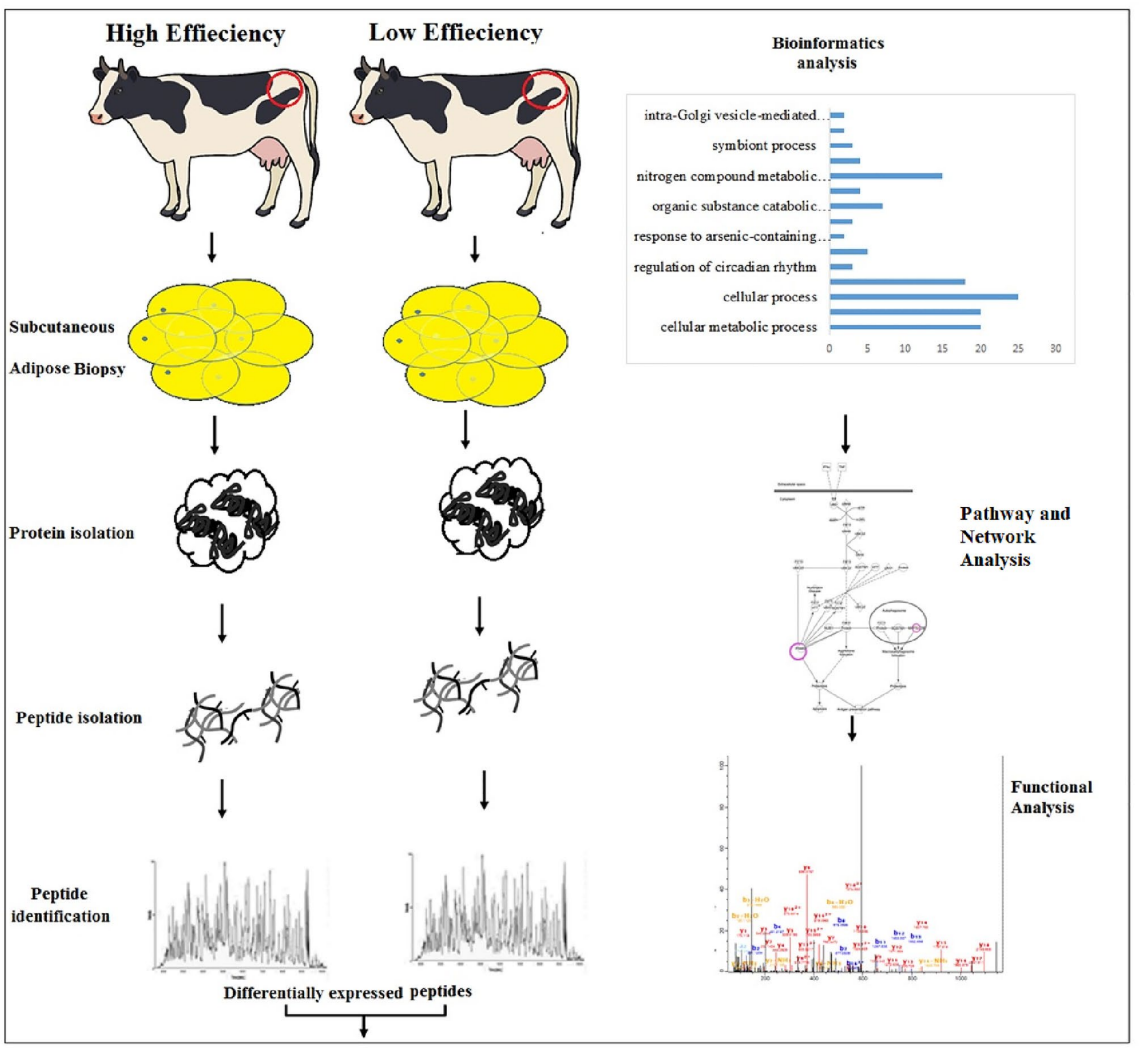
Workflow used to study proteomic analysis of AT in HEF versus LEF dairy cows. AT was isolated from HEF and LEF groups, extracted proteins from samples. Proteins were digested and peptides were identified for the differential expression. Bioinformatic analysis and network analysis was performed to identify the FE related pathways generated by IPA (Qiagen). MS/MS spectra analysis confirmed the presence of peptides in the sequences of FE related proteins.

We identified differential proteins in subcutaneous AT of HEF versus LEF and examined the top pathways, networks and functional analysis related to FE (Fig. 1). In the proteomic analysis of AT, 22,600 raw reads were identified in AT of HEF versus LEF dairy cows, all together 22,600 unique spectra were matched to 17,949 peptides and additionally mapped to 2260 unique proteins (supplementary file 1). Using a threshold of FC ± 1.5 and *P* ≤ 0.05, 101 (4.5% of total proteins) were differentially abundant peptides (DAPs; 43 increased abundances and 58 reduced abundance) in HEF versus LEF AT (supplementary file 2).

### Analysis of Differentially abundant proteins

**Figure 2:**
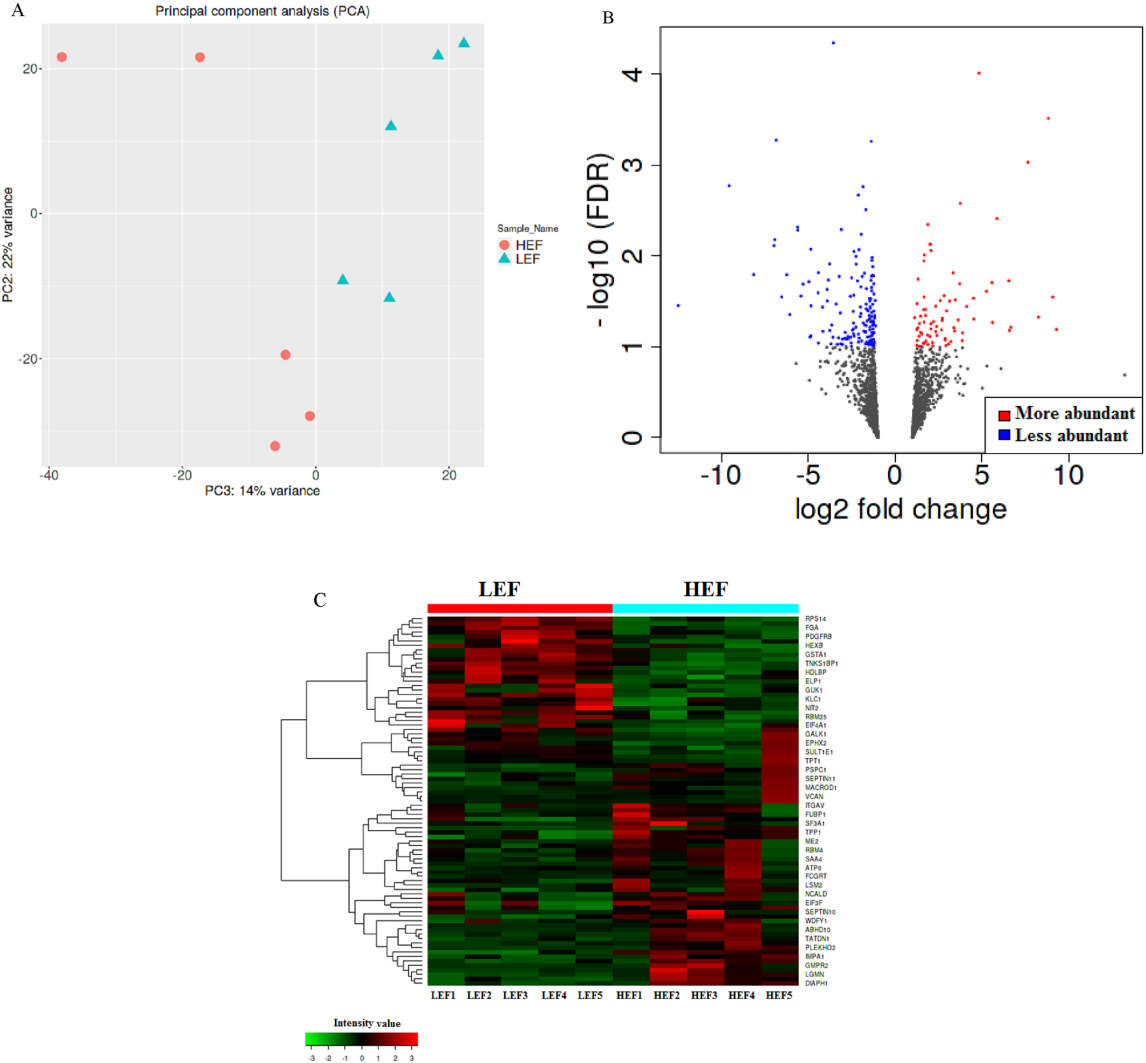
(**A**) Principle component analysis of HEF versus LEF dairy cows measured by IDEP9.1 server showing 14% variance. LEF samples represented in red circles whereas HEF samples represented in green triangles. (**B**) Volcano plot analysis of HEF versus LEF dairy cows generated by IDEP9.1 server with P value (< 0.05) on Y-axis and FDR (± 1.5) on X-axis. Each dot represents one protein and red color indicates more whereas blue color indicates less abundant proteins in AT. (**C**) Heatmap of AT analyzed by IDEP9.1 server where low peptide intensity represented by green color whereas high peptide intensity was measured in red color. Each cow in the study is numbered and represented in columns.

As stated above, the differential abundance analysis of proteome using P-value (0.05) and fold change (± 1.5) in HEF versus LEF AT showed variations among these groups (Fig. 2). The principle component analysis of HEF and LEF AT showed a variance of 14% change in the differential abundance and was well separated; indicating the change in the abundance of peptides in HEF versus LEF samples (Fig. 2A). The increased and decreased abundance proteins were identified by Volcano plot (pi-score, *P* < 0.05; Fig. 2B).

The abundances of several proteins that are potentially related to FE; such as transferrin (TF; FC = 78.35, *P* = 0.02), platelet derived growth factor receptor beta (PDGFRB; FC = 9.08, *P* = 0.03), septin 10 (FC = 8.82, *P* = 0.0003), and RNA-binding motif protein 25 (RBM25; FC = 8.26, *P* = 0.05), were increased in AT of HEF compared to LEF dairy cows. The heat map of 101 DAPs showed overall change (increased and decreased) in abundance of proteins in AT of HEF cows compared to LEF (Fig. 2C).

### The gene ontology (GO) analysis of differentially abundant peptides (DAPs)

**Figure 3:**
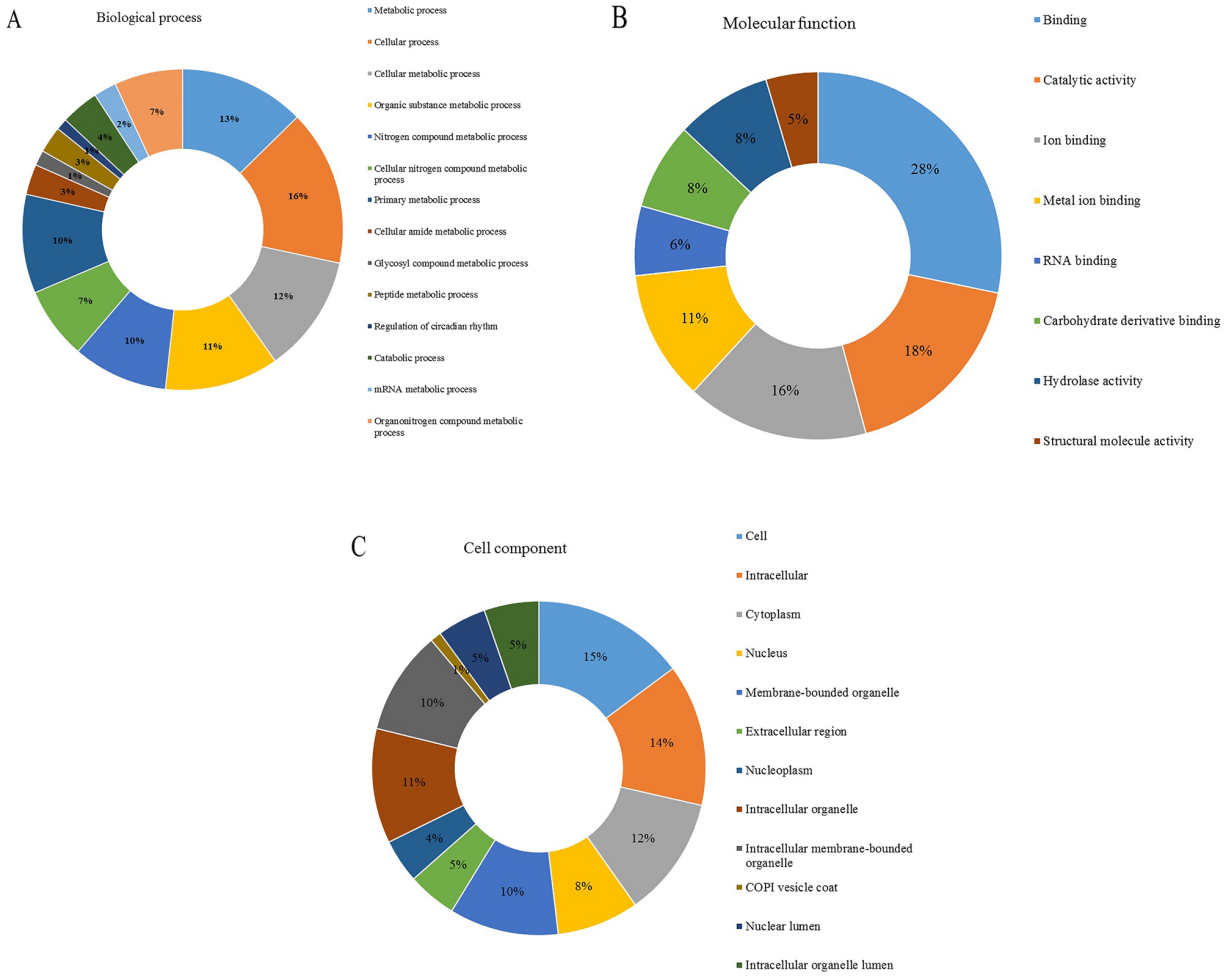
GO analysis of differentially abundant peptides of HEF versus LEF. (**A**) A pie chart of Biological process category (**B**) A pie chart of Molecular function category; (**C**) A pie chart of Cell component category.

The GO analysis of the identified DAPs in HEF versus LEF dairy cows is shown in Fig. 3. In the biological process group, DAPs were assigned to cellular process (16% proteins), metabolic process (13% proteins) cellular metabolic process and organic substance metabolic process (12% and 11% proteins). Noticeably, some DAPs were assigned to nitrogen compound metabolic process (10% proteins), primary metabolic process (10% proteins) and cellular nitrogen compound metabolic process (7% proteins) (Fig. 3A). For the molecular function group, the DAPs involved in GO terms like binding (28% proteins), catalytic activity (18% proteins) ion binding (16.0% proteins) and metal ion binding (11% proteins) and also related to RNA binding (6% proteins), carbohydrate derivative binding (8% proteins), hydrolase activity (6% proteins) and structural molecule activity (5% proteins) (Fig. 3B). In the cellular component group, DAPs were mainly allotted to cell (16% proteins), intracellular (15% proteins), cytoplasm (13% proteins), intracellular organelle part (11% proteins), membrane bound and intracellular membrane bound organelle (10% proteins). Notably, a small number of the DAPs were located in the extracellular region (4% proteins), extra cellular space (3.0% proteins) and ribonucleo protein (2% proteins) (Fig. 3C).

### Regulatory effects of DAPs

**Figure 4:**
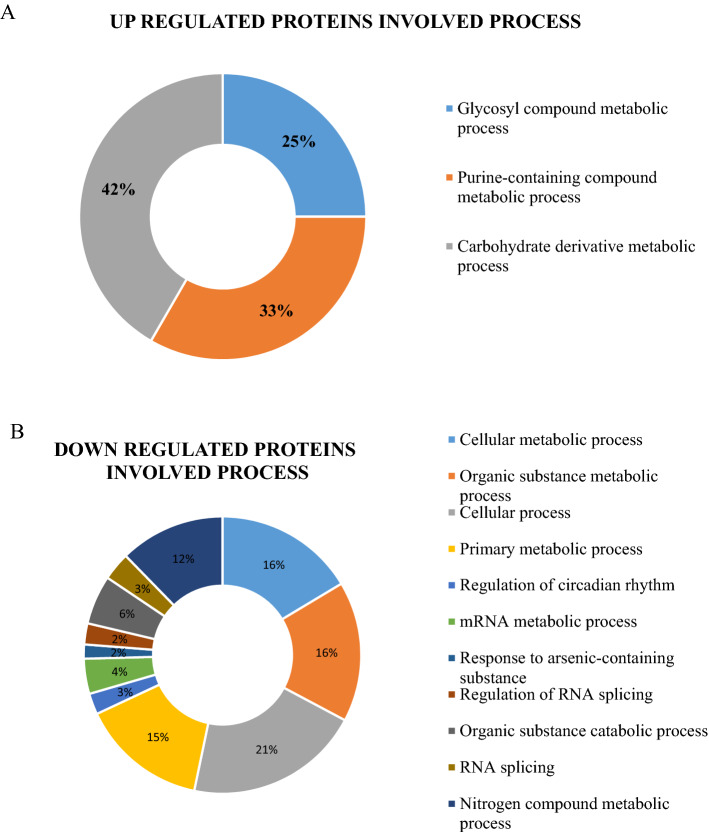
GO terms of up and down regulating process of HEF versus LEF groups. (**A**) A pie chart of upregulated peptides involved process; (**B**) A pie chart of down regulated peptides involved process.

The AT proteome in the HEF group were enriched with proteins of glycosylic, purine and carbohydrate metabolic process related to FE. Notably, twelve up regulated proteins were enriched in the GO term metabolic process. Among these five proteins were up regulated in carbohydrate metabolic process Abhydrolase domain containing 10 (ABHD10); Mevalonate diphosphate decarboxylase (MVD); Guanylate kinase 1 (GUK1); MACRO domain containing 1 (MACROD1) and ATP synthase F0 subunit 6 (MTATP6), four in purine compound containing metabolic process (MVD, GUK1, MACROD1 and ATP6) and three in glycosyl compound containing metabolic process (ABHD10, GUK1 and MACROD1) (Fig. 4A). In contrast, the decreased proteins were principally enriched in cellular metabolic process, organic substance metabolic process, cellular process and primary metabolic process (83 decreased proteins). Additionally, some proteins that were decreased were connected to the nitrogen compound metabolic process, cellular nitrogen compound metabolic process and macromolecule metabolic process (Fig. 4B). Importantly, decreased proteins were associated with the regulation of RNA splicing and cellular catabolic process. In addition, twenty proteins connected to the GO term “cellular metabolic process and organic substance metabolic process” were decreased in HEF versus LEF AT. The GO term “cellular process” was enriched in twenty-five down-regulated proteins. Importantly, 18 proteins with decreased abundance were involved in primary metabolic process. Furthermore, 15 decreased proteins were connected to nitrogen compound metabolic process and 13 decreased proteins were related to macromolecule metabolic process.

### Canonical pathway analysis of DAPs

**Figure 5:**
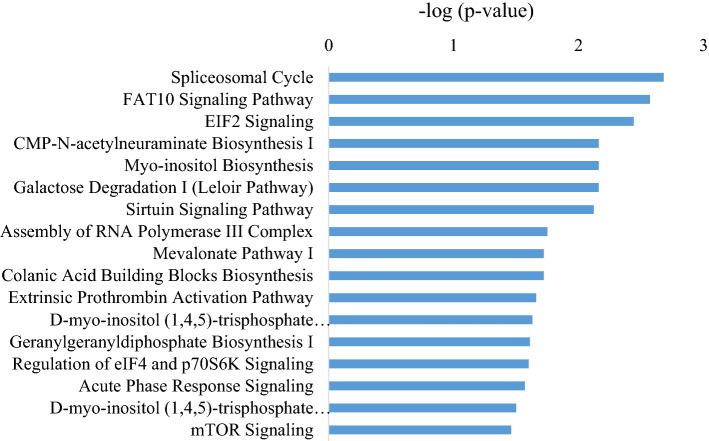
IPA analysis of top canonical enriched pathways in AT of HEF versus LEF groups. Top 18 canonical pathways related to feed efficiency of HEF versus LEF dairy cows on Y-axis and—log (*P*-value) on the X-axis.

The DAPs were analyzed by ingenuity pathway analysis (IPA) software for the top canonical pathways. Figure 5 shows the top canonical pathway enrichment analysis of DAPs affected by RFI in AT of HEF vs LEF dairy cows; the top enriched pathways were related to Spliceosomal Cycle (− log P = 2.68), FAT10 signaling pathway (− log P = 2.57), EIF2 signaling pathway (− log P = 2.44), Sirtuin signaling pathway (− log P = 2.12), Regulation of eIF4 and p70S6K Signaling (− log P = 1.6), Acute phase response signaling pathway (− log P = 1.57; based on 2 increased DAPs) and mTOR Signaling (− log P = 1.46). Other pathways include the NRF2-mediated Oxidative Stress Response (− log P = 0.55), Oxidative Phosphorylation (− log P = 0.85), Xenobiotic Metabolism PXR Signaling Pathway (− log P = 0.63), LXR/RXR Activation (− log P = 0.80) and Protein Ubiquitination Pathway (− log P = 1.26) were identified in HEF vs LEF dairy cows. In the analysis, two decreased DAPs (splicing factors—SF3A1, SF3B2) were enriched in the pathway of Spliceosomal Cycle. In contrast, 1 decreased [Proteasome 26S subunit, non-ATPase 9 (PSMD9)] and 1 increased [Microtubule associated protein 1 light chain 3 beta (MAP1LC3B)] DAPs were enriched in FAT10 signaling pathway. In addition, 2 decreased [Eukaryotic translation initiation factor 4A1 (EIF4A1); Ribosomal protein L27 (RPL27)] and 1 increased [Eukaryotic translation initiation factor 3 subunit F (EIF3F)] DAPs were enriched in the EIF2 signaling pathway, while 1 decreased (SF3A1) and 2 increased (MAP1LC3B, MT-ATP6) DAPs were enriched in the sirtuin signaling pathway. Also 1 increased DAP (MVD) was enriched in Acute phase response signaling pathway.

### Functional analysis of the common DAPs

The commonly abundant proteins were further analyzed in FE functions by IPA software (Table 2). Splicing factors (SF3A1 and SF3B2) downregulated in the pathways related to Spliceosomal Cycle, Assembly of RNA polymerase and Sirtuin signaling pathway. TF upregulated in eight pathways including mTOR signaling, FXR/RXR Activation, LXR/RXR Activation, Ferroptosis Signaling Pathway, Iron homeostasis signaling pathway, Clathrin-mediated Endocytosis Signaling, HIF1α Signaling and Hepatic Fibrosis Signaling Pathway. The decreased protein levels of DnaJ homolog subfamily B member (DNAJB11) was related to Protein ubiquitination pathway, NRF2-mediated Oxidative Stress Response, and Aldosterone Signaling in Epithelial Cells. Importantly, Eukaryotic translation initiation factor 3 subunit F was upregulated and Eukaryotic initiation factor 4A1 was downregulated in four pathways of energy metabolism EIF2 Signaling, Acute Phase Response Signaling, mTOR Signaling and Insulin Secretion Signaling Pathway.

**Table 2:** Downregulated/Upregulated proteins in canonical pathways related to feed efficiency.

Downregulated/Upregulated proteins in canonical pathways related to feed efficiency	Fold change	*P*-value
Splicing factor 3a subunit 1 (SF3A1)	− 3.85	0.018
Splicing factor 3b subunit 2 (SF3B2)	− 3.10	0.005
Transferrin (TF)	78.35	0.022
DnaJ heat shock protein family (Hsp40) member B11 (DNAJB11)	− 3.92	0.020
Eukaryotic translation initiation factor 4A1 (EIF4A1)	− 4.85	0.035
Eukaryotic translation initiation factor 3 subunit F (EIF3F)	2.07	0.008
Ribosomal protein L27 (RPL27)	− 1.55	0.040
Microtubule associated protein 1 light chain 3 beta (MAP1LC3B)	3.75	0.002
ATP synthase F0 subunit 6 (MT-ATP6)	3.72	0.002
Proteasome 26S subunit, non-ATPase 9 (PSMD9)	− 4.97	0.019

### Molecular network analysis

**Figure 6:**
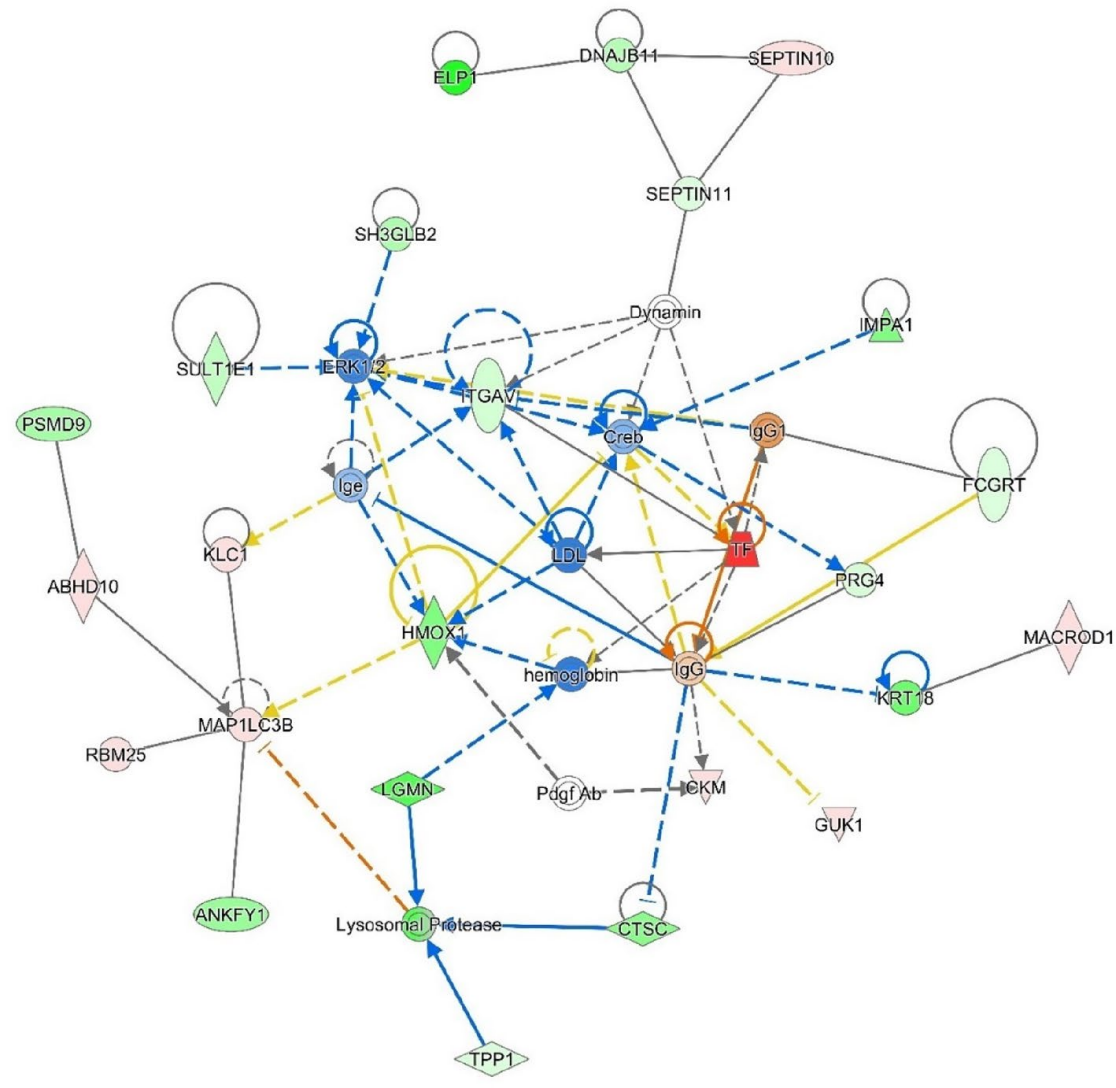
Molecular interaction of Transferrin (TF) involved energy metabolism network generated by IPA (Qiagen). The upregulated peptides represented in red color and green color indicates downregulation. Direct interaction represented in lines and indirect interaction shown in dotted lines.

The direct relationships between molecules related to FE enriched canonical pathways are shown in Fig. 6*.* Thus, in Ingenuity “energy metabolism” was the significant biofunction related to the DAPs, followed by “carbohydrate metabolism” and “protein ubiquitination”. The associated interaction network map in terms of these aforementioned molecules is shown in Fig. 6. The network linked to energy metabolism including FE, in which more abundant TF connecting to low-density lipoprotein (LDL) activation. Also the network showed some proteins related to FE including DNAJB11, serine/threonine protein kinase (ERK12), Integrin subunit alpha V (ITGAV), and Ribosome binding motif 25 (RBM25).

### Gene expression in AT of HEF versus LEF

Several genes involved in energy metabolism related mechanisms including transcription/translation [Signal transducer and activator of transcription 2 (*STAT2),* DExD-Box Helicase 39A *(DDX39A)* and Ribosme binding motif 39 (*RBM39*)] and protein transport (*ITGAV*) were selected for qRT-PCR analysis (Table 3). A decrease in the expression of *RBM39* was observed in adipose of HEF dairy cows compared to LEF group (*P* = 0.05). The relative gene expression of *ITGAV* tended to be higher in HEF versus LEF AT (*P* = 0.07; Table 3). No difference in the relative expressions of *DDX39A,* and *STAT2* were observed between groups (Table 3).

**Table 3:** Relative gene expression in adipose of high or low- efficient dairy cows.

RQ	LEF	HEF	*SEM*	*P*-value
*DDX39A*	1.00	0.77	0.01	0.43
*ITGAV*	1.00	0.78	0.01	0.07
*STAT2*	1.00	1.03	0.01	0.13
*RBM39*	1.00	0.37	0.11	0.05

### MS peptide identification and structure prediction of transferrin (TF)

**Figure 7:**
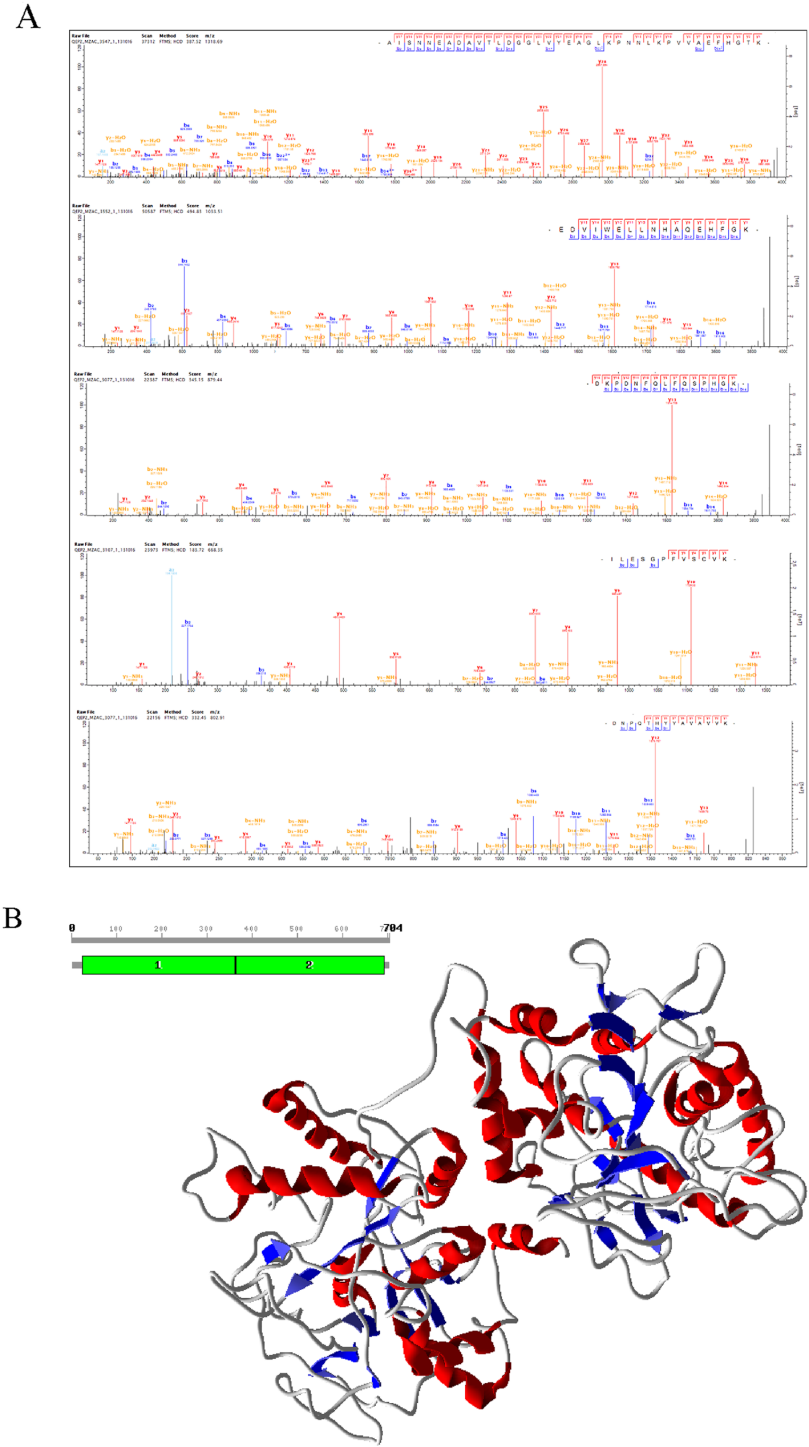
Identification of Transferrin (TF) peptides and structure. (**A**) MS spectra of top abundant TF peptides identified by LC–MS/MS; (**B**) Modelled structure of TF generated by MODELLER9V7 software showing sheets in blue color and helices in red color.

Among the identified proteins linked to FE canonical pathways and network, TF was focused and peptide fragments were identified by the MS/MS approach. The MS/MS spectra of five abundant peptides of TF was shown in Fig. 7A. The peptides were matched in the TF sequence of *Bos taurus* and the sequence collected from Uniprot database used to develop a three dimensional structure. TF contains Peptidase S60, transferrin lactoferrin -like domain (25–693 AA). The model was generated by using a template structure of diferric porcine serum transferrin (PDB code: 1H76_A) from *Sus scrofa* collected from protein data bank as a result of BLAST search where the template showed maximum similarity (74.5%) with TF from *Bos taurus*. The alignment between these sequences showed conserved regions in both and the TF structure was optimized by molecular dynamics and validated using Ramachandran plot server using PROCHECK program (Fig. 7B).

## Discussion

Feed efficiency is an essential aspect of maintaining sustainable dairy herds. Any improvement in feed efficiency will reduce land and water uses and lower greenhouse gasses emitted from the lower number of cows. Identifying high and low efficient lactating cows requires direct measurement of individual DMI, which is accompanied by massive investments in infrastructure and labor. Therefore, establishing protein biomarkers associated with efficiency can be used to identify low or high efficient cows, and this may be utilized to improve efficiency via breeding. Furthermore, understanding the physiological factors involved in efficiency may improve efficiency via better nutrition practice and management. In the present study, we aimed to examine whether differences in the feed efficiency, as measured by RFI values, will be reflected in changes in protein abundance in the subcutaneous AT of mid-lactation dairy cows. Indeed, we found 101 differential proteins in AT of cows that differ in their RFI, from which several proteins related to carbohydrate metabolism were more abundant in the AT of HEF versus LEF cows. In addition, AT of HEF cows showed enrichment of metabolic pathways and networks that can be related to FE.

The AT, as the major energy reserve in mammals, plays a crucial role in the successful establishment and support of lactation^25^. The AT proteome is extremely useful for better understanding the complex factors that control overall metabolism in high-yielding dairy cows^26^. Because of AT's metabolic importance, we hypothesized that variations in FE and energy expenditure (as measured by RFI) would be reflected in the AT proteome. Understanding the key biological processes that lead to RFI variation is important to understand the trait's biological basis. The RFI values of the dairy cows in this study revealed a significant difference between HEF versus LEF groups, and identified DAPs within the AT of mid lactation cows. Similarly, other studies identified canonical pathways including Aldosterone Signaling in Epithelial cells, EIF2 Signaling and NRF2 mediated Oxidative Stress Response connected to RFI in bull hepatic tissue^27,28^. Together, this demonstrates that molecular biomarkers in peripheral tissues can be related to FE in cattle.

The data of this work highlights the connection between RFI and the proteomic networks linked to subcutaneous AT function, and suggest a relationship between RFI and the Spliceosomal Cycle, FAT10 signaling pathway, EIF2 signaling pathway, Sirtuin signaling pathway, Regulation of eIF4 and p70S6K Signaling, Acute phase response signaling pathway and mTOR Signaling in AT. The role of these pathways in protein processing, oxidative response, and cell signaling in the AT are likely to be processes that varies FE. Among the DAPs, Splicing factors (SF3A1 and SF3B2) were downregulated in the pathways related to Spliceosomal Cycle, Assembly of RNA polymerase and Sirtuin signaling pathway. Splicing factors involved in pre-mRNA splicing and translation mechanisms affecting energy metabolisms like carbohydrates, proteins and vitamins^29^.

TF was upregulated in eight pathways in HEF versus LEF AT, among them LXR/RXR Activation^30^, FXR/RXR Activation^31^ and HIF1α Signaling^32^, that were reported previously in relation to FE. The decreased protein levels of DnaJ homolog subfamily B member (DNAJB11) was related to NRF2-mediated Oxidative Stress Response, Protein Ubiquitination Pathway and Aldosterone Signaling in Epithelial Cells, and these pathways plays an important role in FE^32,33^. Importantly, Eukaryotic translation initiation factor 3 subunit F was increased while Eukaryotic initiation factor 4A1 was decreased in four pathways of energy metabolism. Previous studies related to proteomic experiments have showed the relation between DAPs of FE and RFI^34,35^. However, this is the first report of AT tissue of mid lactation cows with an up-regulation of TF, EIF3F, MAP1LC3B and MT-ATP6 of low versus high RFI.

In the modeling of TF, we observed 56 peptides in TF of HEF vs LEF within the subcutaneous AT. The significant role of TF in canonical pathways with *P*-value (*P* < 0.02) and high fold change (FC ± 78.35) in HEF versus LEF AT suggests that TF should be further examined in future studies on FE. TF peptides were matched to sequence and predicted structure containing these peptides by homology modeling. The identification of peptides and structure of TF may help to compare and study the biomarkers of FE.

We found significant pathways underlying FE variation in AT of mid lactation cows using GO enrichment analysis. These analyses highlight the role of AT in the low RFI dairy cows (HEF). The association of RFI and AT has been studied before, and oxidative stress has been related to variations for FE among dairy cows^36^. Some of the DAPs identified in this study were related to oxidative phosphorylation of AT tissue in efficient dairy cows. Another study showed a relation between decreased respiration and increased ROS in less efficient animals^36^. Furthermore, it has been previously demonstrated that HEF have a stronger capacity to adjust oxidative stress conditions^37^, whereas, low efficient animals have increased oxidative stress, shown by their high anti-oxidative activities^38^.

From the results of the GO and IPA analysis of this study, protein processing, oxidative response and cell signaling in the AT are likely to be mechanisms that influence FE variation in dairy cows. The splicing factors SF3A1 and SF3B2 related to splicesomal cycle were down regulated in AT regulating RFI was reported earlier in FE at both protein and transcriptome levels^39^. The protein DNAJB11 that was downregulated in aldosterone signaling pathway was identified as one of the important other canonical pathways in the AT tissue of HEF cows. The adrenal glands release aldosterone, which is important for fluid homeostasis. This aldosterone signaling pathway previously has been linked to FE of inefficient animals^40^, and a GWAS investigation of cattle found this pathway to be connected to feed conversion ratio variation^41^. DNAJB11 belongs to DNAJ-B family and is linked to proper folding of proteins^42^. DNAJB11, in particular, also plays an important role in protein digestion and metabolism, as well as acting as a co-chaperone with HSPA5^43^. In previous studies, DNAJB11 already has been related to FE^33,40^. In agreement with the current study, other researchers^33,40,44^ observed that animals having high FE, have a lower abundance of DNAJB11. These findings may suggest that FE animals have a stronger capacity for controlling cellular mechanisms including protein metabolism. DAPs were also implicated in network analysis as contributing to variance in RFI phenotype^18^. Similarly, in our study, it has been shown that dairy cows with increased FE have a high abundant of TF and lower abundant of *ITGAV, DNAJB11,* Cathepsin (CTSC) in the network analysis. In energy metabolism network generated by IPA, different proteins involved in regulation of FE including DNAJB11, SEPTIN 10, ITGAV, HMOX1, LDL, ERK12, TF, CTSC, ABHD10, MAPILC3B and LDL.

In the present study, the relative gene expression of RBM39 was lower in HEF than in LEF AT. RBM39 is a protein present in the nucleus of cells, among other spliceosome proteins, and is involved in steroid hormone receptor replication and alternative splicing^45^. Because there is no specific information in the literature about the role of RBM39 in AT, its role in AT of efficient cows is unclear. Integrins are proteins that are part of the extracellular matrix in the AT^46^. In this study, we found a tendency for an increase in ITGAV expression in HEF versus LEF AT. It was demonstrated that knocking down ITGAV causes a decrease in proliferation and an increase in adipogenic differentiation of AT stem cells^46^, implying that ITGAV has a detrimental effect on adipogenic cell differentiation. Because we found a tendency for an increase in ITGAV expression in the AT of the efficient cows, this could be linked to a decrease in adipogenic differentiation of the AT stem cells in the AT. This could be consistent with a possible decrease in adipogenesis related to the decrease we observed in STAT2 expression in the AT of efficient cows, as the transcription factors STAT proteins (signal transducers and activators of transcription) are expressed during the adipogenesis process^47^.

In conclusion, we found that differences in feed efficiency between mid-lactating dairy cows were reflected in changes in the proteome of the subcutaneous AT. Therefore, the findings from the present study suggest that the subcutaneous AT may be a source of protein biomarkers of FE in dairy cows. We found differential proteins in HEF that are related to metabolic and other processes that could be related to FE in adipose of dairy cattle. Specifically, the higher abundance of TF and lower abundance of DNAJB11 in AT could be related to FE and needs further investigation as molecular biomarkers in dairy cows.

## Methods

### Animals and experimental design

The Volcani Center Animal Care Committee approved the study’s experimental procedure (approval numbers IL 362-0546), and the experiment was carried out in accordance with standards and regulations. The study is reported in accordance with ARRIVE guidelines. The research was carried out at Israel’s Volcani Center experimental farm in Rishon Lezion. This experiment was part of a larger experiment, and details about the cows used and experimental design and procedures were reported in our previous paper^48^. Briefly, a total of 155 lactating Holstein cows were sampled for feed efficiency for over 4 cycles, 8 weeks each, while receiving the same total mixed ration (TMR)^48^. The cows were characterized for efficiency according to two parameters: residual feed intake (RFI) and energy corrected milk (ECM, kg/d)/Dry matter intake (DMI, kg/d) based on data collected from a period of 35 days, according to a previous study^49^. RFI was calculated based on actual intake, as measured daily, and by predicted intake that was calculated according to NRC (2001) equation: Predicted DMI (kg,d) = [(a × ECM) + (b × BW^c)] × (1−e^(−d × (DIM/7 + e))); while a = 0.36, b = 0.123, c = 0.73, d = − 0.22, e = 5.67, according to regression model based on Volcani’s herd intake and production data collected from previous study^50^. ECM yield (kg/d, of standard milk containing 3.5% fat, 5% lactose, and 3.5% protein with energy value of 0.714 Mcal/kg milk (NRC 2001)) was calculated as: milk yield (kg/d) × {[0.3887 × milk fat (%)] + [0.2356 × (milk protein (%)] + [0.1653 × milk lactose (%)]}/3.1338^51^. Cows were classified as high efficient or low efficient according to RFI and ECM/DMI measurements based on the data from all 4 cycles, and then cows that were within the top or low 20% of RFI values from all cows were classified as high efficient (HEF; top 20% RFI values) or low efficient (LEF; lowest 20% RFI values). Following the classification of cows to HEF and LEF, a subgroup of 10 healthy cows (5 HEF and 5 LEF) were selected for adipose sampling and analysis. The sample size for proteome analysis was based on earlier studies in dairy cows^52^. At the day of biopsy, the body condition score of the cows (scale 1–5) was examined by a single technician.

Blood samples were collected from the 10 cows selected for AT sampling at 0700 h (after morning milking and 3 h before fresh feed delivery). The blood samples were collected by coccygeal venipuncture into vacuum tubes containing lithium heparin (Becton Dickinson, Cowley, UK), and tubes were immediately placed in ice. Centrifugation of blood was performed at 4000×*g* for 15 min and then placed at − 80 °C pending analysis. Plasma samples were analyzed for concentrations of glucose, insulin and BHBA. The plasma concentrations of glucose in plasma were analyzed by using Cobas C111 Autoanalyser (Roche Holding GmbH, Grenzach- Wyhlen, Germany). The intra- and interassay coefficient of variance (CV) for the glucose assay were 2.5 and 2.2%, respectively. Plasma insulin concentrations were determined by radioimmunoassay (MP Bio-medicals, Solon, OH, USA). The intra- and interassay CV for the insulin assay were 6.9 and 4.8%, respectively. The concentration of 3-hydroxybutyrate and dehydrogenase in plasma was evaluated using a Ranbut D-3-Hydroxybutyrate kit (Randox, Crumlin, UK), for the quantity of BHBA in the sample. An optical density reader was used to evaluate the samples at 340 nm, and the results were calibrated for BHBA levels^53,54^ The intra- and interassay CV for the BHBA assay were 1.3 and 1.6%, respectively.

### Collection of adipose tissue

Subcutaneous AT biopsies from the fat pad around the pin bones were taken from 5 HEF and 5 LEF cows at 109 ± 6 DIM. Each subgroup of cows (HEF and LEF) included 2 primiparous and 3 multiparous cows, with an average age of 3.75 years for HEF and 3.78 years for LEF (SEM = 0.56, *P* = 0.98), and average lactation number was 2.4 in HEF and 2.0 in LEF (SEM = 0.47, *P* = 0.67). The AT biopsy was conducted as previously described^23^. In short, the biopsy site, a 5 × 5 cm area of skin on one side of the pin bone, was prepared by clipping, washing and sterilizing. Cows were sedated with an intramuscular administration of 1 mL of 2% Sedaxylan (xylazine base, 20 mg/mL; Eurovet Animal Health, AE Bladel, the Netherlands). The biopsy site was anesthetized by an 8 mL subcutaneous injection of 2% lidocaine HCl (Esracain 2%, 200 mg per 10 mL; Rafa Laboratories Ltd, Israel). A 1.5–2.5 cm scalpel incision was made, under aseptic conditions, through the skin and subcutaneous tissues. In each cow, four samples of approximately 40 mg of fat tissue were captured using tweezers and scissors, tissue samples were then washed with saline followed by snap freeze in liquid nitrogen and then stored at − 80 °C. Immediately after the biopsy, the wound was washed again with 70% alcohol, closed with staples, and covered with an aerosol bandage. Incision sites were inspected daily for 1 week, kept clean, and treated with an aerosol bandage spray if necessary. Staples were removed after 7–10 d.

### Sample preparation for proteomic analysis

The bicinchoninic acid assay was used to determine protein concentration in each AT sample. The samples were tryptic digested and after lysing samples in 1 ml SDT lysis buffer (4% SDS, 100 mM Tris pH 7.6, DTT 100 mM) for 6 min at 95 °C centrifugation (16,000×*g*,10 min) was done to remove cell debris. 50 µg of the supernatant was mixed with 200 µl of urea buffer I (8.0 M urea in 0.1 M Tris–HCl pH 8.0), loaded onto a 30-kDa molecular-weight-cutoff filter (vivacon 500, VN01H22, Sartorius, Göettingen, Germany), and centrifuged for 30 min at 14,000 g, followed by one wash with urea buffer I and again centrifuged for 30 min at 14,000 g. Iodoacetamide was then added to the filter, which was incubated for 10 min before being centrifuged for 20 min at 14,000 g. Using 200 micro liters of ammonium bicarbonate, two washes were performed. The samples were incubated at 37 °C overnight with trypsin (1 g) in 40 L ammonium bicarbonate.

### Data processing and analysis

The raw data was processed and proteins were measured using intensity-based label-free proteomics^55^. The Maxquant 16.6.0 was used to import raw data. Following this, isotopic clustering and feature detection based on peak volume in retention time, m/z, and intensity space were performed. The data were compared to the *Bos taurus* sequences in UniprotKB (http://www.uniprot.org), which were supplemented with common laboratory-contaminating proteins. The carbamidomethylation of cysteines was set as the fixed modification, and the oxidation of methionines was set as the variable modification. Protein grouping and quantification were conducted using an in-house script^55^. Unless the protein was detected with only one or two peptides, protein quantification was predicated on the three most abundant peptides per protein. The total ion current was used to normalize the data. To analyze the data's overall integrity and look for outlier samples, principal component analysis was performed.

### Bioinformatic analysis

Qiagen Ingenuity Pathway Analysis (IPA, Qiagen Redwood City, CA) was used to find the most important pathways for proteins that were differentially abundant at *P* ≤ 0.05 and FC ± 1.5. For functional enrichment analysis, expression data was imported into the IPA Software. IPA predicts regulatory networks linked with an expression list of genes using data from databases and assigns a statistical Z-score to each network. This Z-score estimates how the network will change as a result of the given gene expression profile. Prediction algorithms and the hypergeometric distribution method were used to identify canonical pathways and functional regulatory networks of upstream regulators. The significance level for pathway and network analysis was set at *P* < 0.05 and *P* < 0.01, respectively. The GO terms including biological process, cell component and molecular function were performed with String version 10.5 considering *P*-value < 0.05 as significant (*P*-adjust < 0.05), and the *Bos taurus* genome as background. Principal components analysis (PCA), volcano plot and Heat map were plotted with IDEP 9.1 packages.

### Modeling of structure for Transferrin (TF)

TF structure containing the identified peptides was modeled using protein sequence collected from UNIPROT database from *Bos taurus* (Uniprot_KB Accession Id: G3X6N3). For domain identification, the TF sequence in FASTA format was submitted to SBASE (pongor.itk.ppke.hu) server, and to find out the related protein structure; the predicted domains were searched by BLAST (blast.ncbi.nlm.nih.gov) against PDB^56^. Using the default parameters in ClustalX^57^, the sequence of template aligned with the target sequence^58^. Homology modeling was done by using MODELLER9V7 software to construct the initial model of TF^59^. This software generated fifty models for TF, and the least energy model was selected depending upon the lesser objective function. Later, by molecular dynamics simulation, the protein was stabilized by adding hydrogens to the three-dimensional structure. With the help of the NAMD 2.8 and CHARMM27 force field, MD simulations of the predicted model were performed^60^. In molecular dynamics studies, the structure of TF with lesser Root Mean Square Deviation (RMSD) is achieved, and to examine the Stereochemical quality of protein structures; it is then figured out by Ramachandran plot, using PROCHECK server^61^. Later the environment profile is checked using structure evaluation server ERRAT^62,63^.

### Quantitative real-time PCR of AT samples in HEF versus LEF

Gene expression of the samples were measured in AT^64^. The RNeasy lipid tissue micro kit was used to homogenize using metal bead to 40 mg of AT samples in 1 mL of lysis solution for RNA extraction (Qiagen, Hilden, Germany). The RNA purity was assessed using a Nanodrop, and the 260/280 ratio of the RNA quality was found to be greater than 1.85. A cDNA reverse transcription kit was used to make first-strand cDNA (Applied Biosystems, Foster City, CA). Real-time PCR was used to detect specific mRNA transcripts quantitatively using a StepOnePlus equipment (Applied Biosystems) and the SYBR green PCR mix (Invitrogen, Carlsbad, CA). We investigated at the expressions of FE-related genes such as DDX39A, ITGAV, STAT2, and RBM39. The primers were listed in supplementary Table 1. Data were standardized for the quantity of the reference gene GAPDH mRNA in AT samples, and primers were validated before use. After investigating many candidate genes in AT (UXT, BRPS2, EIF4E, GAPDH, and -actin), the GAPDH expression levels were found to be the most consistent among the AT samples, and this gene was chosen as a reference gene. The delta-delta CT (relative quantity, RQ) of each gene was employed for statistical analysis; the data were divided by the average RQ of the LEF group for each gene.

### Statistical analyses

Comparisons between the two efficiency groups were summarized by day with respect to parameters RFI, ECM/DMI, DMI, and yield of milk, milk component, fat, protein and lactose, and ECM. JMPpro-15.0 software was used to analyze the data using a mixed model (SAS Institute Inc., 2016), with treatment as fixed effect, cow as random effect with day as repeated measures of cow as subject using AR(1) structure. For comparisons of means between groups, Student's T tests were performed. For each cow, the average DIM, parity, average daily gain (ADG), and initial BW were analyzed by Student’s T tests to compare mean values between groups. Shapiro–Wilk’s goodness of fit test was used to ensure normal distribution (*P* = 0.681).

After logarithmic transformation, the Student's t-test, two-tailed, equal variance, was used to identify significant differences across the biological replica for proteomic data. The ratio of arithmetic means of the case and control samples was used to calculate fold changes. After logarithmic transformation, differences in gene expression were evaluated using SAS's GLM procedure.

### Institutional review board statement

The experimental protocol for the study was approved by the Volcani Center Animal Care Committee (approval numbers IL 362-0546), and the experiment was performed in accordance with relevant guidelines and regulations.

## Supplementary Information

Supplementary Information 1.

Supplementary Information 2.

Supplementary Information 3.

Supplementary Information 4.

## Data Availability

The mass spectrometry proteomics data have been deposited to the ProteomeXchange Consortium via the PRIDE partner repository with the dataset identifier PXD029328. The source data underlying all experimental results (Figures and Supplementary data) are provided as a Source Data file. The authors declare that all other data supporting the findings of this study are available within the paper and its Supplementary information files.
