# Supplementary material for: Proteomic analysis of adipose tissue revealing differentially abundant proteins in highly efficient mid-lactating dairy cows
Source: Sci Rep. 2022 Jun 13;12:9721. doi: 10.1038/s41598-022-13964-x (PMC9192684; doi:10.1038/s41598-022-13964-x)
Supplement: Supplementary file 4 — Supplementary Information 4. [file 41598_2022_13964_MOESM4_ESM.pdf]

Proteomic analysis of adipose tissue revealing differentially abundant proteins in highly efficient mid-lactating dairy cows

Yehoshav A. Ben Meir[1,†], Jayasimha R. Daddam[1,†], Gitit Kra[1], Hadar Kamer[1], Yuri Portnick[1], Yishai Levin[2], Maya Zachut[1,*]

[1]Department of Ruminant Science, Institute of Animal Sciences, Agricultural Research Organization, Volcani Center, Israel

[2]The Nancy and Stephen Grand Israel National Center for Personalized Medicine, Weizmann Institute of Science, Rehovot 7610001, Israel

[†]Equal contribution

*Corresponding author: mayak@volcani.agri.gov.il

Supplementary Table 1: List of primers used for mRNA expression in the AT samples

Gene	GenBank accession no.	Sequence 5`>3`[2]
**DDX39A**	NM_001034752.1	F:GTGAAGCACTTCGTGTTGGA<br>R:ATCATGCACTGCTTCTCGTG
**ITGAV**	NM_174367.1	F:CGTTCACACTTTGGGTTGTG<br>R:GGTCCACAGGAGAGACCTCA
**STAT2**	NM_001205689.1	F:AAGTTCACCGTCCGAACAAG<br>R:TTGGGAGAATCCCTGTCAAC
**RBM39**	NM_001206504.1	F:TCAGAGGCCGCTACAGAAGT<br>R:GGACTTTTGCTTCGAGAACG
